# The stage-specific regulation and role of root-knot nematode SWEET genes

**DOI:** 10.1371/journal.ppat.1014161

**Published:** 2026-05-06

**Authors:** M. Willow H. Maxwell, Bharat Rohilla, Jasper Chippendale, Chris A. Bell

**Affiliations:** 1 Faculty of Biological Sciences, School of Biology, University of Leeds, Leeds, United Kingdom; 2 Cell and Molecular Sciences, The James Hutton Institute, Invergowrie, United Kingdom; University of California Davis, UNITED STATES OF AMERICA

## Abstract

The root-knot nematode *Meloidogyne incognita* is a globally significant plant parasite that causes substantial crop losses. While pre-parasitic juveniles rely on innate energy reserves, later life stages acquire nutrients from host plants through specialized feeding structures. SWEET (*Sugars Will Eventually be Exported Transporter*) genes exhibit a conserved sugar transporting ability across all kingdoms of life, yet their function in plant-parasitic nematodes remains underexplored. Here, we functionally characterise the SWEET gene family in *M. incognita*, revealing their critical and stage-specific roles in nematode development and parasitism. We demonstrate that *Mi-SWEET*s segregate into two functional groups: those that facilitate mobility and invasion in motile juveniles (*Mi-SWEET2, 4*) and those support nutrient uptake during feeding (*Mi-SWEET3, 5, 7*). Although temporally distinct, all SWEET genes localise to the intestine, suggesting a conserved role in mediating sugar flux. Knockdown of *Mi-SWEET2* and *Mi-SWEET4* reduced root invasion, while silencing *Mi-SWEET3, 5,* and *7* impaired post-invasion growth, highlighting the varied roles of this large gene family across different life stages. Yeast complementation assays revealed distinct substrate preferences among Mi-SWEETs, aligning with the metabolic needs of different life stages. The transcription factor HBL1, a key regulator of nematode dietary responses, was found to control the expression of *Mi-SWEET3* and is itself regulated through interaction with the post-transcriptional regulatory microRNA *let-7*. Our findings provide new insights into the metabolic adaptations and energy utilisation of plant-parasitic nematodes and outline a microRNA - transcription factor - target gene regulatory network. These findings have broader relevance given the fundamental importance of the regulation of resource transportation in plant-pathogen interactions.

## Author summary

Root-knot nematodes are soil-dwelling parasites that threaten global food production by invading plant roots and diverting nutrients for their own growth. These nematodes must switch between two very different metabolic strategies: juveniles rely on stored energy reserves to move through the soil, while later developmental stages feed directly from specialised feeding sites within plant roots. How nematodes control the movement of sugars, one of their key energy sources, has remained largely unknown.

In this study, we identify and characterise a family of sugar transporter genes, called SWEETs, in the root-knot nematode *Meloidogyne incognita*. We show that different SWEET genes are switched on at different life stages and fulfil distinct roles in mobility and feeding. We demonstrate that some SWEETs are essential for successful root invasion, while others support nematode growth once feeding has begun. We uncover a regulatory network involving a transcription factor and a conserved microRNA that controls expression of a feeding-related SWEET gene. Together, our findings reveal how nematodes manage their energy resources.

## Introduction

Crop production is of utmost importance for future food security and the quantity and stability of yields is heavily burdened by parasites. Plant-parasitic nematodes are a large contributor to yield losses, predicted to cause yield reductions of 12.3% per annum (equating to $157 billion/ year) [1,2]. The root-knot nematode *Meloidogyne incognita* can infect more than 3000 plant species, including many economically important crops [1]. These losses are a result of the nematodes biotrophic lifestyle that necessitates their feeding from plant tissue, ultimately reducing plant vigour. In brief, second-stage juvenile (J2) root-knot nematodes hatch from eggs and navigate towards the root based on the perception of root-exuded signals. During this motile stage, the nematode relies upon its innate lipid reserves and does not feed [3], with body lipid content directly correlating with nematode infectivity [4,5]. Little is known about the utilisation of these innate resources during nematode migration, however presumably these lipids are metabolised to provide energy for nematode propulsion [6]. The nematode’s lipid content decreases from hatching until it begins feeding [7], at which point the lipid content increases again from the influx of plant material [8,5]. *Meloidogyne incognita* genome analysis has identified orthologues related to the gluconeogenesis pathway that are up-regulated in juvenile stages of this, and other diverse, plant-parasitic nematodes [9,10]. By utilising these innate energy reserves, the nematode migrates intercellularly to the vascular cylinder where feeding begins. Feeding is a complex process where the juvenile nematode induces redifferentiation of host cells into large, multinucleate cells with enhanced metabolic activity. These nutrient dense cells sustain the nematode through its life cycle [1]. Ingested plant-products are metabolised within the nematode intestine via endoglucanases [11,12], proteinases [13,14], and detoxification enzymes [15]. The nematode intestine is relatively ill-defined and requires further investigation to ascertain the pathways involved in the utilisation of host compounds.

Sugars Will Eventually be Exported Transporter (SWEET) are an increasingly studied family of sugar transporters that are widespread across bacteria, metazoan and plants, with a conserved sugar transporting activity [16,17]. In animals, their copy number can vary between organisms, for example humans have a single copy whereas *C. elegans* has seven genes [18] and the number within plant-parasitic nematodes may range from two to potentially over ten [19]. Although plant SWEETs appear to have conserved substrates based on their clade [20], our understanding of animal SWEETs is lacking. *Caenorhabditis elegans* SWEET1 transports glucose or galactose when expressed in HEK293T cells or *Xenopus* oocytes, respectively [18]. A genome-wide RNAi screen suggested a role of *C. elegans* SWEET1 in the regulation of lipid metabolism, perhaps through a feedback mechanism linked to the utilisation of glucose [21]. *Globodera pallida SWEET3* is part of a negative feedback mechanism controlled by the transcription factor Gp-HBL1, which is regulated by nematode dietary intake [19]. Consequential regulation of *Gp-SWEET3* expression impacts nematode development and hexose intake [19]. Further understanding of how these damaging pests transport and utilise their energy resources would assist efforts aimed at restricting their feeding from hosts. Additionally, a lack of further data on this gene family, which is widely researched in plants, is required to understand its role and importance throughout animals.

In addition to transcriptional regulation, gene expression in nematodes is extensively shaped by microRNAs (miRNAs), small non-coding RNAs that post-transcriptionally regulate mRNA stability and translation [22]. In *C. elegans*, the conserved miRNA *let-7* directly targets the 3′ untranslated region of the transcription factor *HBL-1*, forming a regulatory circuit that couples developmental timing to transcriptional outputs [23]. Conservation of miRNAs and their targets across nematodes suggests that similar miRNA-transcription factor interactions may operate in parasitic species to modulate developmentally regulated gene expression [24].

Here, we present data that describes the differential roles of root-knot nematode SWEET genes throughout development. We characterise the gene family both spatially and temporally as well as determine the saccharide substrates of different SWEET clades, outlining the role of root-knot nematode SWEETs. Additionally, we propose a regulatory pathway for a SWEET gene, consisting of a transcription factor and animal-conserved miRNA. The ability of a single gene family (SWEET) to have a prominent role in multiple life stages indicates a conserved function of core, yet distinct, genes that are present in all plant-parasitic nematodes.

## Results

**Fig 1:**
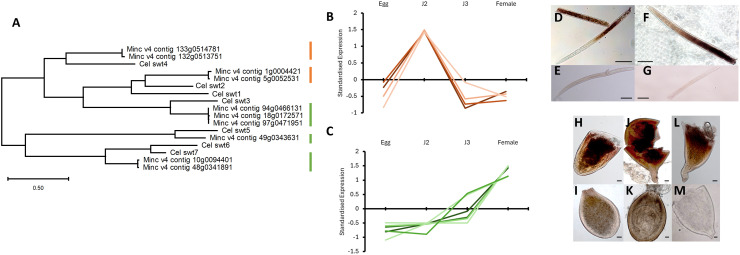
Temporal and spatial characterisation of *Meloidogyne incognita* SWEET genes. **A)** Maximum likelihood phylogenetic tree of SWEET amino acid sequences from *M. incognita* and *Caenorhabditis elegans.* Orange and green columns indicate the expression-based cluster of *M. incognita* genes, as visualised in B & **C.** B, **C)** The expression clusters of *M. incognita* SWEET genes; second-stage juvenile (B) and female (C) expression peaks. D-M) *In situ* hybridisation was conducted to visualise the expression of each SWEET gene using homeologue conserved probe sequences. *Mi-SWEET2* (D, E) and *Mi-SWEET4* (F, G) were localised in second-stage juveniles. *Mi-SWEET3* (H, **I)**, *Mi-SWEET5* (J, K) and *Mi-SWEET7* (L, M) were localised in females. E, G, I, K, M represent sense strand controls. Scale bars = 50 um.

The amino acid sequences of the seven *Caenorhabditis elegans* SWEET genes were used to identify *M. incognita* orthologues via reciprocal BLAST. Alignment and phylogenetic analysis revealed ten *M. incognita* SWEETs (Fig 1A); orthologues for five of the seven *C. elegans* SWEETs. Multiple homeologues were identified in *M. incognita* for certain SWEET genes: *Mi-SWEET2* (two; Minc_v4_contig_1g0004421, Minc_v4_contig_5g0052531), *Mi-SWEET3* (three; Minc_v4_contig_18g0172571, Minc_v4_contig_97g0471951, Minc_v4_contig_94g0466131), *Mi-SWEET4* (two; Minc_v4_contig_132g0513751, Minc_v4_contig_133g0514781), *Mi-SWEET5* (one; Minc_v4_contig_49g0343631) and *Mi-SWEET7* (two; Minc_v4_contig_10g0094401, Minc_v4_contig_48g0341891). Although *Ce-swt1* and *Ce-swt6* did not yield a direct orthologue via reciprocal BLAST searches, they exhibit high amino acid sequence identity to *Ce-swt2* (51%) and *Ce-swt7* (65%) respectively.

Analysis of the expression profiles of all ten *M. incognita* genes across the nematode’s development indicated that there were two expression-based classes. Four genes had highest relative expression in second-stage juveniles (Fig 1B), whereas six genes had greatest expression in females (Fig 1C) (TPM provided in S1 Table; confirmatory qRT-PCR of each distinct SWEET gene is presented in S1 Fig). Genes within the two expression-based classes were found to cluster within phylogenetic tree analysis (Fig 1A). Subsequent analysis focused on the distinct SWEET genes rather than their homologues, due to over 97% amino acid sequence similarity between homeologues (S2 Table).

**Fig 2:**
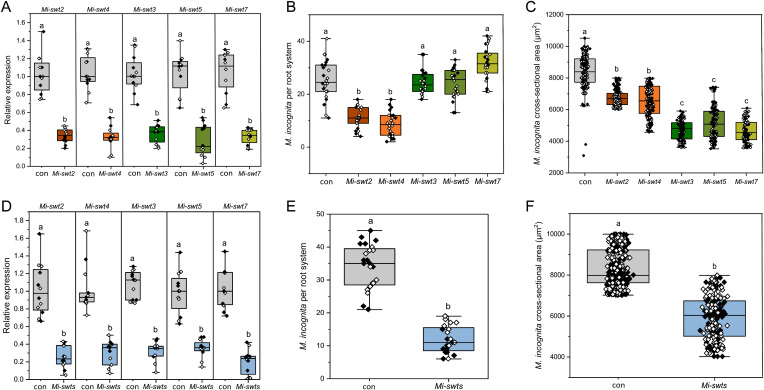
The impact of *Mi-SWEET* knockdown on nematode parasitism. **A)** Knockdown of *Mi-SWEET2, 3, 4, 5* or *7* via DsiRNA treatment. Three DsiRNA targeting different locations of each transcript were used for each gene and applied to second-stage juveniles for 24 hrs. DsiRNA with no *M. incognita* targets were applied to control nematodes (grey). The expression of the target genes were quantified by qRT-PCR using Elongation Factor 2 as a reference gene and displayed relative to control nematodes. N = 6, two biological replicates. **B)** Root invasion of *Mi-SWEET* knockdown nematodes 14 dpi on tomato roots. N = 12, two biological replicates. **C)** The cross-sectional area of a maximum 20 nematodes per root system was measured at 14 dpi. 12 root systems per biological replicate (2); N = 206-480. **D)** SWEET gene knockdown similar to A, but a combined DsiRNA treatment targeting five *Mi-SWEET* genes (2, 3, 4, 5, 7). **E)** Root invasion of second-stage juveniles post-knockdown of five *Mi-SWEET*s 14 dpi on tomato roots. **F)** The cross-sectional area of nematodes with reduced *Mi-SWEET* gene expression was measured 14 dpi, as in **C.** Twelve root systems per biological replicate (2); N = 225-490. Letters denote statistical significance in all panels using two-sample t test P < 0.01 in A, D, E & F, and Oneway-ANOVA with Tukeys post host analyses P < 0.01 in B & **C.** White and black coloured datapoints indicate biological replicates. In A, B & C orange boxes indicate genes with expression in J2s whereas green boxes indicate genes expressed in females.

*In situ* hybridisation indicated that *Mi-SWEET2* and *Mi-SWEET4* were expressed within the intestine of J2 nematodes (Fig 1D and 1F). Expression of *Mi-SWEET3*, *Mi-SWEET5* and *Mi-SWEET7* were localised within the intestine of female nematodes (Fig 1H, 1J, and 1L). Exposure of J2s to DsiRNA complementary to *Mi-SWEET 2, 3, 4, 5* or *7* resulted in gene knockdown to 26–46% of that observed in control nematodes treated with DsiRNA containing a scrambled sequence with no known nematode targets (Fig 2A; two-sample t-test P < 0.01). Knockdown of *Mi-SWEET2* & *4* resulted in a reduced number of nematodes invading the root system, when quantified at 14 dpi (Fig 2B; Oneway-ANOVA with Tukeys post host analyses P < 0.01). The nematodes that did infect the root system had a slightly reduced cross-sectional area compared to control nematodes at 14 dpi (Fig 2C; Oneway-ANOVA with Tukeys post host analyses P < 0.01). Knockdown of *Mi-SWEET3, 5* & *7* resulted in no effect on nematode root invasion (Fig 2B), however greatly reduced surface area compared to control or *Mi-SWEET2* and *4* knockdown nematodes at 14 dpi (Fig 2C; Oneway-ANOVA with Tukeys post host analyses P < 0.01). A combined treatment of all *SWEET*-targeting DsiRNA was performed and resulted in knockdown of all five SWEET genes to 23–37% of that observed in control nematodes, 24 hours after treatment (Fig 2D; two-sample t-test P < 0.01). Combined knockdown yielded reduced invasion (Fig 2E; two-sample t-test P < 0.01) as well as reduced cross-sectional area at 14 dpi (Fig 2F; two-sample t-test P < 0.01), compared to control nematodes.

**Fig 3:**
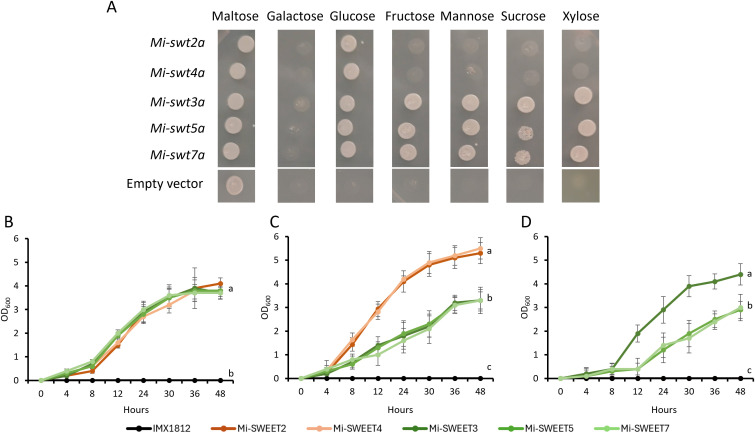
Yeast complementation assay to determine the substrates of Mi-SWEETs. **A)** IMX1812 yeast transformed with pRS410:*Mi-SWEET2, 3, 4, 5* or *7,* and grown on minimal SD base agar supplemented with amino acids and either 2% maltose, galactose, glucose, fructose, mannose, sucrose or xylose. Yeast were grown for three days at 32°C. All images are taken from the same hexose-supplemented plate. Empty vector transformants were used as controls. **B)** The growth rate of each transformant was monitored over 48 hours on maltose **(B)**, glucose (C) and sucrose (D) by OD600 measurements. Yeast were grown on minimal SD base supplemented with amino acids and 2% maltose, glucose or sucrose. Letters denote statistical significance between the growth curves of different yeast strains using two-way repeated measures ANOVA with Tukeys post hoc analyses, P < 0.01.

A hexose transporter-deficient yeast strain (IMX1812) was complemented with *Mi-SWEET*s to determine their substrate specificity and assist clarification of their potential role/s at different stages of parasitism. IMX1812 transformed with the empty vector (pRS410) was unable to consume the provided hexose carbon sources other than maltose, as expected (Fig 3A) [25]. Yeast transformed with *Mi-SWEET2 or 4* grew on media supplemented with maltose (control), or glucose (Fig 3A). *Mi-SWEET3, 5* or *7* expressing yeast were able to grow on media supplemented with maltose (control), glucose, fructose, mannose, sucrose or xylose. No *Mi-SWEET*s rescued IMX1812 growth on galactose.

The growth rate of each *Mi-SWEET* transformant was measured due to shared hexose substrates. *Mi-SWEET2* and *4* transformed yeast showed a greater growth rate on glucose compared to *Mi-SWEET3, 5* and *7* (Fig 3C; two-way repeated measures ANOVA with Tukeys post hoc analyses P < 0.01). Growth rates were measured on sucrose-supplemented media due to potential variance in colony growth observed between *Mi-SWEET3, 5* and *7.* Here, *Mi-SWEET3* transformed yeast exhibited a greater rate of growth compared to all other *Mi-SWEET* transformants (Fig 3D; two-way repeated measures ANOVA with Tukeys post hoc analyses P < 0.01).

**Fig 4:**
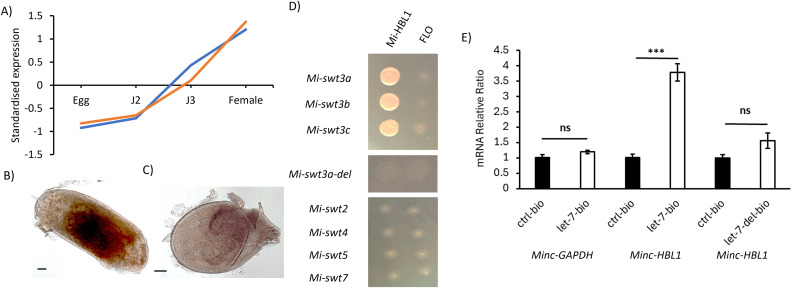
The regulation network of *Mi-SWEET3.* **A)** The expression pattern of *Mi-HBL1* homeologues throughout development. B & **C)** *In situ* hybridisation of *Mi-HBL1* in parasitic stages using a homeologue conserved probe sequence. A sense strand probe was used as a control **(C)**. Scale bars = 50 um. **D)** Yeast-one hybrid assays between Mi-HBL1 transcription factor protein and 1kb promoters of *Mi-SWEET3a* (Minc_v4_contig_94g0466131)*, 3b* (Minc_v4_contig_18g0172571) and *3c* (Minc_v4_contig_97g0471951), and *Mi-SWEET2* (Minc_v4_contig_1g0004421)*, 4* (Minc_v4_contig_132g0513751)*, 5* (Minc_v4_contig_49g0343631) or *7* (Minc_v4_contig_10g0094401). FLO, a DNA-interacting protein, was used as a control. Yeast were grown on media lacking histidine and supplemented with 45mM 3-AT. E) 3’-biotinylated *let-7* mimics were synthesised and used to pulldown nematode mRNA. *let-7* or *let-7-del* (with a deleted seed sequence (gagguag)) were incubated with nematode lysate and purified by streptavidin-beads. *Mi-HBL1* and *Mi-GAPDH* (as a control gene) were quantified by qRT-PCR. The C_t_ value of *Mi-HBL1* or *Mi-GAPDH* in the *let-7-bio* or *let-7-del-bio* pull-downs were displayed as a relative ratio to the C_t_ value of the same gene in pulldowns using random 3’ biotinylated RNA (ctrl) [26]. Each qRT-PCR was performed three times on each sample, with four biological replicates. Asterisks denote significance between bars at P < 0.001, two-sample t-test.

The *Globodera pallida* transcription factor HBL1 represses *Gp-SWEET3* expression [19], therefore the orthologue, *Mi-HBL1* was identified; Minc_v4_contig_34g0272541 & Minc_v4_contig_2g0020911. Homeologues share 98% amino acid identity to each other and 58% identity to *G. pallida* HBL-1. *Mi-HBL1* was found to be expressed increasingly throughout development (Fig. 4A) and within intestinal regions of parasitic stages (Fig 4B & 4C). *Mi-HBL1* was expressed in yeast along with “bait” vectors containing the 1kb promoter regions of the three homeologues of *Mi-SWEET3 (*named *a, b* and *c*)*.* Growth on selection media indicated an interaction between Mi-HBL1 protein and the *Mi-SWEET3* homeologue promoters (Fig 4D). No interactions were observed between the Antirrhinum flowering related protein FLO [27], confirming no unspecific promoter-protein interactions. Mi-HBL1 did not interact with the 1kb promoter regions of homeologues of *Mi-SWEET2, 4, 5* or *7* (Fig 4D)*.* The *Mi_SWEET3* promoter lacking the HBL-1 binding motif predicted in *C. elegans* and *G. pallida* (([A/T]TTTTTTC; [19,23]) did not interact with HBL1 (Fig 4D).

To advance our understanding of plant-parasitic nematode gene regulation, specifically of SWEET genes, we investigated the interaction between *Mi-HBL1* and the microRNA *let-7,* due to the known regulation of *HBL-1* by *let-7* in other nematode species [23]. *Mi-let-7* (ugagguaguagguuguauaguu; [24]) has a predicted complementary site located 361 bp downstream of the *Mi-HBL1* stop codon. A 3′-biotinylated *Mi-let-7* mimic was used to pulldown the *Mi-HBL1* transcript from nematode cell lysate. There was significantly greater *Mi-HBL1* mRNA recovered in the *let-7-bio* pulldowns compared with pulldowns using a biotinylated random RNA control (Fig 4E; P < 0.001, two-sample t-test). *Mi-GAPDH* mRNA levels were similar between *let-7-bio* and control pulldowns, indicating a lack of non-specific RNA enrichment. Deletion of the *let-7* seed sequence (gagguag) in the biotinylated probe (*let-7-del-bio*) abolished significant *Mi-HBL1* mRNA pulldown (Fig 4E; P = 0.062, two-sample t-test).

## Discussion

Root-knot nematodes must utilise various resources to fuel their invasion of host roots, subsequent development and production of a second generation. This fuel is derived from innate lipid reserves and then subsequently bolstered by plant lipids and sugars acquired during feeding from host tissue. Our data show that root-knot nematodes have a suite of SWEET genes that are important throughout nematode development due to their role in facilitating the transport of hexoses across the intestinal lumen. There are two classes of SWEET genes that are expressed either during the motile J2 stage, thereby potentially facilitating the utilisation of gluconeogenesis products derived from lipid granules, or during feeding, where they may facilitate the influx of host sugars. Here, we illustrate how members of this gene family are expressed within the same tissue yet can impact different stages of the parasite’s life cycle. Additionally, we outline a miRNA - transcription factor - target gene regulatory network of a feeding-related SWEET gene. Overall, determining how these pathogens utilise their energy stores represents a potential target to manipulate to aid their control, and sheds light on the inter-kingdom role of SWEETs.

SWEET genes have been identified in all kingdoms of life, with a conserved sugar transport activity [16]. Despite plant genomes containing numerous SWEET genes with described structure and functions, animals generally have a reduced complement that are relatively ill-defined [17]. Nematodes appear to contain additional SWEET genes compared to other animals with *C. elegans* containing seven [17] and the number in plant-parasitic nematodes ranging from two (*Pratylenchus coffeae* [19]) to ten (*M. incognita;* this study). The 10 genes identified in *M. incognita* share high similarity to *C. elegans* SWEETs and appear to have multiple homeologues, consistent with its large genome [28,29]. The majority of *M. incognita* SWEETS were expressed uniquely in either motile or feeding stages, with no genes expressed equally throughout development.

Although there is differential temporal expression of *M. incognita* SWEETs, there appears to be conserved localisation within the intestine. A lack of detailed intestinal structural resolution prevents definitive exclusion of expression in overlapping tissues, such as the female reproductive tract or the J2 subventral glands; however, the large amorphous gut in these stages strongly indicates intestinal expression, if not exclusively. This indicates a conserved spatial function, suggesting that they transport sugars out of the intestinal lumen, as proposed for *Globodera pallida* SWEET3 [19]. The J2s that exhibit high expression of *Mi-SWEET4* and *2* do not feed from the plant, therefore their role is potentially to transport sugars derived from innate energy stores out of the intestine for use by muscles. Upon hatching, the J2 has a finite lipid reserve that it relies upon to migrate towards, and enter, the root. This energy store decreases after hatching and only replenishes if the nematode succeeds in feeding from the root [5]. Genome analysis confirms the presence of conserved energy metabolism pathways, such as gluconeogenesis [30]. Anabolised carbohydrates require transportation out of the intestine before they can be utilised by nematode tissues, presenting a potential role for motile-expressed SWEET genes. Temporal and spatial gene expression analyses of *Mi-SWEET4* and *2* suggests that they may be active in transporting the hexose products of lipid metabolism pathways out of the intestine to fuel nematode mobility. *Mi-SWEET3, 5* and *7* were expressed in the intestine of J3s/females, indicating a role in the transportation and utilisation of ingested sugars.

Knockdown of *Mi-SWEET2* or 4 resulted in a reduction in root invasion, whereas knockdown of SWEET genes *3, 5* and *7* (expressed highest in females) resulted in a strong reduction in nematode size. Knockdown of genes that are expressed during migratory stages is known to reduce root invasion [31], whereas knockdown of feeding-related genes has previously shown a reduction in nematode growth rates [19]. Similar to the potato cyst nematode *G. pallida,* Mi-HBL1 appears to interact with the promoter of *Mi-SWEET3* homeologues. Mi-HBL1 did not, however, interact with other *Mi-SWEET* gene promoters, despite shared temporal and spatial expression patterns. This infers that there are multiple transcription factors that regulate intestine gene expression throughout development, contrary to the regulation of gene expression in the subventral [31] and dorsal [32] glands of cyst nematodes. This may suggest the presence of multiple compartments/portions to the intestine that independently express unique sets of genes, rather than the uniform nature of the gland cells. The intestine of the model nematode *C. elegans* consists of a single epithelial tube composed of 20 cells [33]. The lumen contains of microvilli upon which several proteins localise to enable a variety of functions, with dietary import of high importance [34]. Several transcription factors, such as ELT-2, are known to interact to direct expression uniquely throughout the 20 intestinal cells [35,36]. The *in situ* hybridisation imaging undertaken in this study, and others on plant-parasitic nematodes [12,13,37,38], potentially conflate multiple intestinal compartments as a single structure when in fact there are compartment-specific functions. It is possible that some elements of the structure and regulatory networks of the *C. elegans* intestine are conserved in plant-parasitic nematodes, however further work using single-cell RNAseq, laser microdissection or use of cell-type specific markers if genetic transformation of these organisms can be achieved, would greatly advance our understanding of this crucial organ.

To elucidate the difference between motile- and feeding-expressed SWEET genes, we performed a yeast complementation assay using hexose-transporter deficient IMX1812 [25]. This strain was chosen due to the several chromosome rearrangements and instability of EBY.VW4000, which was previously the commonly-used strain for this assay [39]. Whilst all five *Mi-SWEETs* enabled growth of the yeast strain on glucose-supplemented media, *Mi-SWEET2* and *4*, which were expressed in J2s, enabled a greater rate of growth. This could be due to the rapid utilisation of gluconeogenesis products required by juveniles to locate and migrate towards the root, compared to the lengthy feeding processes of sedentary nematodes. Structurally, *Mi-SWEET2* and *4* may be specialised transporters of glucose, enabling their quicker growth on glucose media, as they were unable to transport the other hexoses tested, however structural assays would be required to confirm this. Alternatively, progression of research into broader animal SWEETs would enable sequence-based predictions of function, as is possible with plant SWEET research. *Mi-SWEET3, 5* and *7* had a larger substrate range and were able to rescue IMX1812 grown on media supplemented with glucose, fructose, mannose, sucrose or xylose. Hexoses are enriched within both cyst and root-knot nematode feeding sites [40,41,42], as a result of the up-regulation of cell wall invertases (cleaving sucrose into glucose and fructose) and plant hexose transporters, such as SWEETs [43] within these tissues. The wide substrate range of these SWEETs may reflect the diverse hexoses that are ingested by the nematode from their feeding sites, compared to a potentially reduced hexose range available to mobile J2s. *Mi-SWEET3* exhibited a greater rate of growth on sucrose supplemented media, compared to *Mi-SWEET5* and *7*. Sucrose, the main transported form of photoassimilated carbon in plants [44], accumulates within root-knot nematode feeding sites through the upregulation of sucrose transporters [45,41] as well as symplastic sucrose flux through shifts in plasmodesmatal structure [46]. Similar to our study on *Mi-SWEET3,* certain plant SWEETs are known to transport both mono- and disaccharides, although this is relatively uncommon [47,48,49]. Lack of homology to plant SWEETs prevents our inference from known plant residues that dictate mono or di-saccharide binding [50]. Plant SWEET phylogeny can indicate substrate preference based upon these sequence variations and further work is required to perform similar predictions for animal SWEETs, not just for plant-parasitic nematodes.

The similar hexose transport activity and substrate-specificity of multiple SWEETs suggests potential redundancy, however knockdown of a single gene results in a detrimental nematode phenotype similar to knockdown of five genes. It is possible that RNAi is not a suitably powerful to tease apart the contributions of each SWEET and that knockout mutants are required, however this technology is currently unavailable for these pathogens [51]. Additionally, the transporters may be functioning at their maximum rate despite potential redundancy and any drop below this, by reducing the expression of individual *SWEET*s may have an effect. It is also possible that each SWEET gene is expressed in intestinal tissues that cannot be resolved by the current approach and that they each perform distinct functions that are currently unknown, as discussed earlier.

We investigated the potential role of microRNA as gene regulators in plant-parasitic nematodes, with specific relevance to SWEETs and HBL1. In *C. elegans*, *let-7* regulates the transition from larva-to-adult and is known to reciprocally regulate the expression of *hbl-1* through direct binding within its 3′ UTR [23]. We explored the potential for similar regulation in *M. incognita* using the orthologous *let-7* sequence [24] and identified *let-7*–*Mi-HBL1* 3′ UTR interactions via pulldowns using biotinylated microRNA mimics. *Let-7* functions as a highly conserved developmental regulator across metazoans, controlling cell fate transitions in organisms as diverse as *Drosophila*, vertebrates, and other nematodes [52], which strengthens the likelihood that an analogous regulatory mechanism is retained in plant-parasitic species. Conservation of its potential role in timing the transition to adulthood is supported by the initiation of feeding being a major developmental milestone for this plant-parasitic nematode, which is likely to be supported by developmental regulators. *Let-7* may have been co-opted to regulate parasitism-related processes that are akin to other life-style related developmental milestones in other species. The biotinylated microRNA mimic approach establishes a further component of the SWEET regulatory system in plant-parasitic nematodes and provides a method for exploring the regulatory roles of wider microRNA in these damaging pathogens.

## Methods

### Sequence analysis

*Meloidogyne incognita SWEET* and *HBL1* genes were identified by reciprocal BLAST using the unzipped *M. incognita* v4 [29,53] and *G. pallida* Newton genome assemblies (PRJNA702104). Amino acid sequences were aligned via CLUSTAL [54] before construction of a maximum likelihood phylogenetic tree using IQ-TREE [55]. The life stage expression of *M. incognita* genes were obtained from life-stage RNA sequencing data [56] mapped to the v4 genome [29]. Life stage gene expression (transcripts per million) were displayed as standardised expression values using z-scores.

### Nematode culture

*Meloidogyne incognita* (VW6) were maintained on tomato plants (‘Ailsa Craig’) growing in compost at 25 °C. Infective second-stage juveniles were extracted by washing the roots, cutting into 3–4 cm lengths and placing in a misting chamber. The humid environment promoted hatching of juveniles from eggs, which were then collected.

### Life stage qRT-PCR

qRT-PCR was conducted on eggs, J2s, J3s and Females to validate RNA-seq analyses on the VW6 population. Eggs were collected by handpicking five egg masses from infected roots, confirming the presence of eggs under a microscope, and then freezing in liquid nitrogen. J2s were collected by placing infected roots in a misting chamber, as described above, and freezing in liquid nitrogen upon collection. To collect J3s and females, the roots of infected tomato cultures were washed and briefly blended (approximately 5 seconds) in a small volume of tap water. The solution quickly was poured through a tower of sieves; 300, 150, 63 and 25 µm. The contents of the 150, 63 and 25 µm sieves were inspected and nematodes were immediately removed by pipette and frozen. Two hundred J3 and female nematodes were collected. This process was repeated five times to have five replicate nematode pools for RNA extraction.

Total RNA was prepared from all nematode samples using an E.Z.N.A Plant RNA Kit according to the manufacturer’s protocol including DNase treatment (Omega Biotek, UK). First-strand cDNA was synthesised from 1 µg RNA using iSCRIPT (BioRad, UK) following the manufacturer’s protocol. Analysis of gene expression was carried out using qRT-PCR with Brilliant III Ultra-Fast SYBR Green Master Mix (Agilent Technologies, CA, USA). Cycle conditions were 95 °C for 30 s and subsequently 40 cycles of 5 s at 95 °C and 10 s at 60 °C. Primer pairs (S3 Table) had amplification efficiencies of 90 – 99%. The 2^-ΔΔCt^ method was used to calculate relative expression of each of the five SWEET genes between eggs and other life stages with two technical replicates for each of the five pools of nematodes [57]. Elongation factor 2 was used as a reference gene [58].

### *In situ* hybridisation

Single-strand digoxygenin-labelled anti-sense DNA probes for *Mi-SWEET* and *Mi-HBL1* were amplified from cDNA fragments by primers provided in S3 Table with DIG DNA labelling mix (Roche, Germany). Primers were designed to target specific genes but homeologue conserved regions, using local and Primer BLAST (NCBI). Sense probe controls were constructed in separate reactions as negative controls. These probes were used for *in situ* hybridisation on J2 or parasitic-stage nematodes to determine spatial expression patterns, as previously described [59].

### RNA interference

DsiRNAs were designed for *Mi-SWEETs* using the design tool supplied by Integrated DNA Technologies available at: https://eu.idtdna.com/pages/products/functional-genomics/dsirnas-andtrifecta-rnai-kits. Each sequence was checked via BLAST to ensure no off-target effects (defined as a sequence that shares >16 base identity to the first 19 bases of the siRNA). Three DsiRNA were designed per target gene.

Six pools of approximately 10 000 M. incognita second-stage juveniles were incubated at room temperature in 100 mM octopamine and either target gene DsiRNA (20 µg) or negative control DsiRNA (20 µg; scrambled sequence with no target in *M. incognita*). The three DsiRNA per gene were combined in a single treatment. Nematodes were incubated for 24 hours and then washed five times in tap water. Approximately 1500 nematodes were removed from each incubation for infection assays whilst the remaining nematodes were split into six aliquots and frozen for RNA extraction.

Total RNA was prepared as described above. Primer pairs (S3 Table). The 2^-ΔΔCt^ method was used to calculate relative expression between control and experimental samples with three technical replicates for each of the six biological replicates [57].

One hundred of the treated nematodes were infected on to tomato plant roots in soil-free pouches, as previously described [60]. The nematodes were distributed between four root tips. Each DsiRNA treatment was infected on to 12 plants. Plants were grown for two weeks. Nematodes were then stained with acid fuchsin for quantification. The cross-sectional area of a maximum 20 nematodes from each of the 12 replicates was quantified by ImageJ [61] The exact number of nematodes measured per treatment varied based on infection rate, with 20 being the maximum measured from a single root system.

This was repeated in its entirety for a second independent replicate containing an additional 12 plants. Data are presented on the same plots but distinguished by coloured datapoints.

### Yeast complementation assay

The hexose transporter-deficient yeast strain IMX1812 (Prof Pascale Daran-Lapujade, TU Delft) was complemented with *Mi-SWEET*s to determine their hexose specificity. *Meloidogyne incognita* SWEET coding sequences were codon-optimised for expression in *Saccharomyces cerevisiae* and synthesised by Genewiz (UK), with additional EagI and SpeI restriction sites. Sequences were cloned into pRS410 (Addgene #11258) using the above restriction sites and T4 DNA ligase (New England Biolabs, USA). Plasmids were transformed into IMX1812 with the “Quick & Easy Yeast Transformation Mix” (Takara, US) on SD Agar Base plates (Takara, US) supplemented with 2% maltose and 500 μg/mL G-418 antibiotic.

To screen for hexose transport activity, IMX1812 transformed with empty pRS410 were used as negative controls. Yeast colonies were picked from plates and grown in liquid medium to OD_600_ 0.6 [62]. Cells were washed twice in water and diluted to OD_600_ 0.1 before 5 μl were plated onto SD plates supplemented with 2% maltose (control), galactose, glucose, fructose, mannose, sucrose or xylose. Plates were incubated at 32 °C for four days before imaging.

To quantify the growth rate of each transformant, the OD_600_ of liquid cultures starting at OD_600_ 0.1 was monitored over three days when grown in different hexose media.

### Yeast-one-hybrid

Yeast one-hybrid assays were conducted to test for transcription factor-promoter interactions by MATCHMAKER One-Hybrid System (Clontech, UK). *Mi-SWEET* 1kb promoter regions were synthesized (Genewiz, UK) and cloned into the bait construct, pHisi, and transformed into yeast (YM4271) with the “Quick & Easy Yeast Transformation Mix” (Takara, US). The *Mi-HBL1* (Minc_v4_ contig_34g0272541) prey construct was prepared by cloning the *Mi-HBL1* CDS in-frame with the GAL4 activation domain in the pGADT7-Rec2 plasmid. pGADT7-Rec2-FLO [27] was used as negative control prey. Prey constructs were transformed into the various *Mi-SWEET* bait yeast strains mentioned above. Yeast were grown on SD/-Leu/-Trp (as a positive growth control condition) and SD/-His/-Leu medium supplemented with 45 mM 3-amino-1,2,4-triazole (3-AT), for assessing interactions. For each test, yeast colonies were grown to an OD_600_ of 0.6 before 5 μl of each culture were spotted onto the appropriate SD plates and incubated at 30 ˚C for three days before imaging.

### microRNA pulldown

To determine potential interactions between candidate *HBL1* regulator *let-7,* a *let-7* mimic was synthesized with a 3’ biotin tag (gagguaguagguuguauaguu-bio) and used according to previous methodology [26]. A random 22-nt biotinylated RNA sequence was also synthesized as a control, as well as *let-7* lacking the predicted seed sequence (uagguuguauaguu-bio; *let-7-del-bio* (lacking gagguag). Approximately 100 000 J2s were homogenised by micropestle on ice in 25 mM Tris-HCl (pH 7.5), 70 mM KCl, 2.5 mM EDTA, 0.05% NP-40, 80 U/mL RNase Inhibitor (Applied Biosystems), and 1 x protease inhibitor cocktail (Roche) before centrifugation at 13 000 x g for 20 mins at 4 °C. The supernatant was transferred to a new tube and biotinylated RNA were added (5 nmoles). The solution was incubated at 4 °C with gentle shaking for 30 mins and then 30 °C with gentle shaking for 1 h. Ten μL Streptavidin magnetic beads (New England Biolabs) were pre-treated with 250 μg RNase-free BSA and 100 μg yeast tRNA in 500 μL of 25 mM Tris-HCl (pH 7.5), 70 mM KCl, 2.5 mM EDTA, and 0.05% NP-40 for 2 h, then washed twice more in the same buffer before adding the cell lysate and microRNA mimics to the beads. The Streptavidin/biotin–miRNA/mRNA complex was spun at 5,000 x *g* for 30 s and washed four times at 4 °C for 5 min using 20 mM Tris-HCl (pH 7.4), 400 mM KCl, and 0.5% NP-40. The biotin–miRNA/mRNA complex was eluted with 100 μL of 20 mM Tris-HCl (pH 7.4), 400 mM KCl, 0.5% NP-40, 5 mM biotin, and 80 U/mL RNase inhibitor at 42 °C for 5 min. RNA was extracted from the eluant and cDNA transcribed (using total extracted RNA rather than a normalised amount), as described previously.

*Mi-HBL1* and *Mi-GAPDH* (as a control gene) were quantified from the cDNA by qRT-PCR, using reaction mixes as described previously alongside primers outlined in S4 Table. The C_t_ value of *Mi-HBL1* or *Mi-GAPDH* in the *let-7-bio* or *let-7-del-bio* pull-downs were displayed as a relative ratio to the C_t_ value of the same gene in pulldowns using random 3’ biotinylated RNA (ctrl) [26]. Each qRT-PCR was performed three times on each sample and averaged for one biological replicate. The pulldown, RNA extraction, cDNA synthesis were performed on four different batches of nematodes to yield four biological replicates.

### Statistics

All data were analysed using OriginPro. Two-sample t-tests were used for pairwise comparisons to assess knockdown of *M. incognita* genes. One-way ANOVA followed by Tukey’s post hoc tests were used to determine difference between individual SWEET knockdown treatments on root invasion and the cross-sectional areas of infected nematodes. Two-sample t-tests were used to assess infection and cross-sectional area of nematodes treated with DsiRNA for all *Mi-SWEET*s compared to control. Growth-rate data from yeast complementation assays were evaluated using two-way repeated-measures ANOVA with Tukey’s post hoc correction to compare strains across time points and carbon sources. For *let-7* pulldown assays, enrichment of *Mi-HBL1* mRNA relative to control pulldowns was analysed using two-sample t-tests. A significance threshold of P < 0.01 (or P < 0.001 where indicated) was applied throughout.

## Supporting information

S1 Table *Meloidogyne incognita* SWEET TPM throughout development. Transcript per million of each discussed SWEET gene in *M. incognita* throughout development.
(DOCX)

S2 Table Percentage identity matrix of *M. incognita* SWEET genes. Matrix generated by Clustal2.1 using amino acid sequences of the *M. incognita* orthologues of *C. elegans* SWEET genes.
(DOCX)

S3 Table *In situ* hybridisation probe and qRT-PCR primers. Primers used to amplify DIG probes for *in situ* hybridisation and for qRT-PCR.
(DOCX)

S4 Table *Mi-HBL1* and *Mi-GAPDG* primers. Primers used in PCR to amplify *Mi-HBL1* DIG probes for *in situ* hybridisation, as well as to amplify *Mi-HBL1* and *Mi-GAPDH* for qRT-PCR and DIG probes.
(DOCX)

S1 Fig qRT-PCR validation of RNA-seq expression profiles of ten *Meloidogyne incognita* SWEET genes. The expression of the target genes were quantified by qRT-PCR using Elongation Factor 2 as a reference gene and displayed relative to nematode eggs for each gene.
(DOCX)

## References

[1] Abad P, Favery B, Rosso M-N, Castagnone-Sereno P. Root-knot nematode parasitism and host response: molecular basis of a sophisticated interaction. Mol Plant Pathol. 2003;4(4):217–24. doi: 10.1046/j.1364-3703.2003.00170.x 20569382

[2] Ikram M, Singh S, Ansari MJ, Islam J, Shariq M, Sulaiman Alharbi R, et al. Evaluation of botanicals for the management of Meloidogyne incognita infecting carrot and volatile nematicidal metabolite profiling. Journal of King Saud University - Science. 2023;35(9):102911. doi: 10.1016/j.jksus.2023.102911

[3] Lu C-J, Meng Y, Wang Y-L, Zhang T, Yang G-F, Mo M-H, et al. Survival and infectivity of second-stage root-knot nematode Meloidogyne incognita juveniles depend on lysosome-mediated lipolysis. J Biol Chem. 2022;298(3):101637. doi: 10.1016/j.jbc.2022.101637 35085555 PMC8861644

[4] Christophers AEP, Patel MN, Benson JA, Saka VW, Evans AAF, Wright DJ. : A rapid field-laboratory bioassay to assess the infectivity of Meloidogyne spp. second stage juveniles. Nematol. 1997;43(1):117–20. doi: 10.1163/004725997x00089

[5] Shivakumara TN, Dutta TK, Mandal A, Rao U. Estimation of lipid reserves in different life stages of Meloidogyne incognita using image analysis of Nile Red-stained nematodes. Nematol. 2019;21(3):267–74. doi: 10.1163/15685411-00003212

[6] Rocha FS, Campos VP, Catão HCRM, Muniz MFS, Civil N. Correlations among methods to estimate lipid reserves of second-stage juveniles and its relationships with infectivity and reproduction of Meloidogyne incognita. Nematol. 2015;17(3):345–52. doi: 10.1163/15685411-00002871

[7] Das S, Wesemael WML, Perry R. Effect of temperature and time on the survival and energy reserves of juveniles of Meloidogyne spp. Agricultural Science Research Journal. 2011;1:102–12.

[8] Jones JT, Haegeman A, Danchin EGJ, Gaur HS, Helder J, Jones MGK, et al. Top 10 plant-parasitic nematodes in molecular plant pathology. Mol Plant Pathol. 2013;14(9):946–61. doi: 10.1111/mpp.12057 23809086 PMC6638764

[9] Choi I, Subramanian P, Shim D, Oh B-J, Hahn B-S. RNA-Seq of Plant-Parasitic Nematode Meloidogyne incognita at Various Stages of Its Development. Front Genet. 2017;8. doi: 10.3389/fgene.2017.00190 PMC5711784 29230237

[10] Huang X, Xu C-L, Yang S-H, Li J-Y, Wang H-L, Zhang Z-X, et al. Life-stage specific transcriptomes of a migratory endoparasitic plant nematode, Radopholus similis elucidate a different parasitic and life strategy of plant parasitic nematodes. Sci Rep. 2019;9(1):6277. doi: 10.1038/s41598-019-42724-7 31000750 PMC6472380

[11] Bell CA, Lilley CJ, McCarthy J, Atkinson HJ, Urwin PE. Plant-parasitic nematodes respond to root exudate signals with host-specific gene expression patterns. PLoS Pathog. 2019;15(2):e1007503. doi: 10.1371/journal.ppat.1007503 30707749 PMC6373980

[12] Fanelli E, Troccoli A, Picardi E, Pousis C, De Luca F. Molecular characterization and functional analysis of four β ‐1,4‐endoglucanases from the root‐lesion nematode P ratylenchus vulnus. Plant Pathology. 2014;63(6):1436–45. doi: 10.1111/ppa.12222

[13] Lilley CJ, Urwin PE, McPherson MJ, Atkinson HJ. Characterization of intestinally active proteinases of cyst-nematodes. Parasitology. 1996;113 (Pt 4):415–24. doi: 10.1017/s0031182000066555 8873479

[14] Neveu C, Abad P, Castagnone-Sereno P. Molecular cloning and characterization of an intestinal cathepsin L protease from the plant-parasitic nematode Meloidogyne incognita. Physiological and Molecular Plant Pathology. 2003;63(3):159–65. doi: 10.1016/j.pmpp.2003.10.005

[15] Espada M, Silva AC, Eves van den Akker S, Cock PJA, Mota M, Jones JT. Identification and characterization of parasitism genes from the pinewood nematode Bursaphelenchus xylophilus reveals a multilayered detoxification strategy. Mol Plant Pathol. 2016;17(2):286–95. doi: 10.1111/mpp.12280 25981957 PMC6638532

[16] Hu Y-B, Sosso D, Qu X-Q, Chen L-Q, Ma L, Chermak D, et al. Phylogenetic evidence for a fusion of archaeal and bacterial SemiSWEETs to form eukaryotic SWEETs and identification of SWEET hexose transporters in the amphibian chytrid pathogen Batrachochytrium dendrobatidis. FASEB J. 2016;30(10):3644–54. doi: 10.1096/fj.201600576R 27411857

[17] Yuan M, Wang S. Rice MtN3/saliva/SWEET family genes and their homologs in cellular organisms. Mol Plant. 2013;6(3):665–74. doi: 10.1093/mp/sst035 23430047

[18] Chen L-Q, Hou B-H, Lalonde S, Takanaga H, Hartung ML, Qu X-Q, et al. Sugar transporters for intercellular exchange and nutrition of pathogens. Nature. 2010;468(7323):527–32. doi: 10.1038/nature09606 21107422 PMC3000469

[19] Maxwell MWH, Causier BE, Chippendale J, Ault JR, Bell CA. Diet-regulated transcriptional plasticity of plant parasites in plant-mutualist environments. Proc Natl Acad Sci U S A. 2025;122(16):e2421367122. doi: 10.1073/pnas.2421367122 40244681 PMC12037023

[20] Eom J-S, Chen L-Q, Sosso D, Julius BT, Lin IW, Qu X-Q, et al. SWEETs, transporters for intracellular and intercellular sugar translocation. Curr Opin Plant Biol. 2015;25:53–62. doi: 10.1016/j.pbi.2015.04.005 25988582

[21] Ashrafi K, Chang FY, Watts JL, Fraser AG, Kamath RS, Ahringer J, et al. Genome-wide RNAi analysis of Caenorhabditis elegans fat regulatory genes. Nature. 2003;421(6920):268–72. doi: 10.1038/nature01279 12529643

[22] Ste-Croix DT, Mimee B. MicroRNAs in plant-parasitic nematodes: what are they and why should we care?. J Nematol. 2025;57(1):20250041. doi: 10.2478/jofnem-2025-0041 41018003 PMC12461141

[23] Roush SF, Slack FJ. Transcription of the C. elegans let-7 microRNA is temporally regulated by one of its targets, hbl-1. Dev Biol. 2009;334(2):523–34. doi: 10.1016/j.ydbio.2009.07.012 19627983 PMC2753757

[24] Wang Y, Mao Z, Yan J, Cheng X, Liu F, Xiao L, et al. Identification of MicroRNAs in Meloidogyne incognita Using Deep Sequencing. PLoS One. 2015;10(8):e0133491. doi: 10.1371/journal.pone.0133491 26241472 PMC4524723

[25] Wijsman M, Swiat MA, Marques WL, Hettinga JK, van den Broek M, Torre Cortés P de la, et al. A toolkit for rapid CRISPR-SpCas9 assisted construction of hexose-transport-deficient Saccharomyces cerevisiae strains. FEMS Yeast Res. 2019;19(1):foy107. doi: 10.1093/femsyr/foy107 30285096 PMC6217715

[26] Yamamoto K, Ito S, Hanafusa H, Shimizu K, Ouchida M. Uncovering Direct Targets of MiR-19a Involved in Lung Cancer Progression. PLoS One. 2015;10(9):e0137887. doi: 10.1371/journal.pone.0137887 26367773 PMC4569347

[27] Causier B, Bradley D, Cook H, Davies B. Conserved intragenic elements were critical for the evolution of the floral C-function. Plant J. 2009;58(1):41–52. doi: 10.1111/j.1365-313X.2008.03759.x 19054363

[28] Abad P, Gouzy J, Aury J-M, Castagnone-Sereno P, Danchin EGJ, Deleury E, et al. Genome sequence of the metazoan plant-parasitic nematode Meloidogyne incognita. Nat Biotechnol. 2008;26(8):909–15. doi: 10.1038/nbt.1482 18660804

[29] Mota APZ, Koutsovoulos GD, Perfus-Barbeoch L, Despot-Slade E, Labadie K, Aury J-M, et al. Unzipped genome assemblies of polyploid root-knot nematodes reveal unusual and clade-specific telomeric repeats. Nat Commun. 2024;15(1):773. doi: 10.1038/s41467-024-44914-y 38316773 PMC10844300

[30] McCarter JP, Mitreva MD, Martin J, Dante M, Wylie T, Rao U, et al. Analysis and functional classification of transcripts from the nematode Meloidogyne incognita. Genome Biol. 2003;4(4):R26. doi: 10.1186/gb-2003-4-4-r26 12702207 PMC154577

[31] Pellegrin C, Damm A, Sperling AL, Molloy B, Shin DS, Long J, et al. The SUbventral-Gland Regulator (SUGR-1) of nematode virulence. Proc Natl Acad Sci U S A. 2025;122(11):e2415861122. doi: 10.1073/pnas.2415861122 40063806 PMC11929438

[32] Eves-van den Akker S, Laetsch DR, Thorpe P, Lilley CJ, Danchin EGJ, Da Rocha M, et al. The genome of the yellow potato cyst nematode, Globodera rostochiensis, reveals insights into the basis of parasitism and virulence. Genome Biol. 2016;17(1):124. doi: 10.1186/s13059-016-0985-1 27286965 PMC4901422

[33] Leung B, Hermann GJ, Priess JR. Organogenesis of the Caenorhabditis elegans intestine. Dev Biol. 1999;216(1):114–34. doi: 10.1006/dbio.1999.9471 10588867

[34] The C. elegans intestine. WormBook. https://www.ncbi.nlm.nih.gov/books/NBK19717/10.1895/wormbook.1.133.1 PMC4780959 18050495

[35] Pauli F, Liu Y, Kim YA, Chen P-J, Kim SK. Chromosomal clustering and GATA transcriptional regulation of intestine-expressed genes in C. elegans. Development. 2006;133(2):287–95. doi: 10.1242/dev.02185 16354718 PMC4719054

[36] Williams RTP, King DC, Mastroianni IR, Hill JL, Apenes NW, Ramirez G, et al. Transcriptome profiling of the Caenorhabditis elegans intestine reveals that ELT-2 negatively and positively regulates intestinal gene expression within the context of a gene regulatory network. Genetics. 2023;224(4):iyad088. doi: 10.1093/genetics/iyad088 37183501 PMC10411582

[37] Shingles J, Lilley CJ, Atkinson HJ, Urwin PE. Meloidogyne incognita: molecular and biochemical characterisation of a cathepsin L cysteine proteinase and the effect on parasitism following RNAi. Exp Parasitol. 2007;115(2):114–20. doi: 10.1016/j.exppara.2006.07.008 16996059

[38] Wang H-L, Cheng X, Ding S-W, Wang D-W, Chen C, Xu C-L, et al. Molecular identification and functional characterization of the cathepsin B gene (Ab-cb-1) in the plant parasitic nematode Aphelenchoides besseyi. PLoS One. 2018;13(6):e0199935. doi: 10.1371/journal.pone.0199935 29958285 PMC6025850

[39] Solis-Escalante D, van den Broek M, Kuijpers NGA, Pronk JT, Boles E, Daran J-M, et al. The genome sequence of the popular hexose-transport-deficient Saccharomyces cerevisiae strain EBY.VW4000 reveals LoxP/Cre-induced translocations and gene loss. FEMS Yeast Res. 2015;15(2):fou004. doi: 10.1093/femsyr/fou004 25673752

[40] Hofmann J, Hess PH, Szakasits D, Blöchl A, Wieczorek K, Daxböck-Horvath S, et al. Diversity and activity of sugar transporters in nematode-induced root syncytia. J Exp Bot. 2009;60(11):3085–95. doi: 10.1093/jxb/erp138 19487386 PMC2718214

[41] Sun L, Lian L, Yang R, Li T, Yang M, Zhao W, et al. Sugar delivery at the tomato root and root galls after Meloidogyne incognita infestation. BMC Plant Biol. 2024;24(1):451. doi: 10.1186/s12870-024-05157-7 38789940 PMC11119304

[42] Wang X, Li S, Zhang X, Gao L, Ruan Y-L, Tian Y, et al. From Raffinose Family Oligosaccharides to Sucrose and Hexoses: Gene Expression Profiles Underlying Host-to-Nematode Carbon Delivery in Cucumis sativus Roots. Front Plant Sci. 2022;13:823382. doi: 10.3389/fpls.2022.823382 35251093 PMC8892300

[43] Zhou Y, Zhao D, Duan Y, Chen L, Fan H, Wang Y, et al. AtSWEET1 negatively regulates plant susceptibility to root-knot nematode disease. Front Plant Sci. 2023;14:1010348. doi: 10.3389/fpls.2023.1010348 36824200 PMC9941640

[44] Lemoine R. Sucrose transporters in plants: update on function and structure. Biochim Biophys Acta. 2000;1465(1–2):246–62. doi: 10.1016/s0005-2736(00)00142-5 10748258

[45] Hammes UZ, Schachtman DP, Berg RH, Nielsen E, Koch W, McIntyre LM, et al. Nematode-induced changes of transporter gene expression in Arabidopsis roots. Mol Plant Microbe Interact. 2005;18(12):1247–57. doi: 10.1094/MPMI-18-1247 16478044

[46] Xu L-H, Xiao L-Y, Xiao Y-N, Peng D-L, Xiao X-Q, Huang W-K, et al. Plasmodesmata play pivotal role in sucrose supply to Meloidogyne graminicola-caused giant cells in rice. Mol Plant Pathol. 2021;22(5):539–50. doi: 10.1111/mpp.13042 33723908 PMC8035636

[47] Büttner M, Sauer N. Monosaccharide transporters in plants: structure, function and physiology. Biochim Biophys Acta. 2000;1465(1–2):263–74. doi: 10.1016/s0005-2736(00)00143-7 10748259

[48] Klemens PAW, Patzke K, Deitmer J, Spinner L, Le Hir R, Bellini C, et al. Overexpression of the vacuolar sugar carrier AtSWEET16 modifies germination, growth, and stress tolerance in Arabidopsis. Plant Physiol. 2013;163(3):1338–52. doi: 10.1104/pp.113.224972 24028846 PMC3813654

[49] Lalonde S, Wipf D, Frommer WB. Transport mechanisms for organic forms of carbon and nitrogen between source and sink. Annu Rev Plant Biol. 2004;55:341–72. doi: 10.1146/annurev.arplant.55.031903.141758 15377224

[50] Han L, Zhu Y, Liu M, Zhou Y, Lu G, Lan L, Wang X, Zhao Y, Zhang XC. Molecular mechanism of substrate recognition and transport by the AtSWEET13 sugar transporter. Proc Natl Acad Sci U.S.A. 2017;114:10089–94. 10.1073/pnas.1709241114 PMC5617298 28878024

[51] Kranse O, Beasley H, Adams S, Pires-daSilva A, Bell C, Lilley CJ, et al. Toward genetic modification of plant-parasitic nematodes: delivery of macromolecules to adults and expression of exogenous mRNA in second stage juveniles, G3 Genes|Genomes|Genetics. 2021;11:jkaa058. 10.1093/g3journal/jkaa058 PMC8022973 33585878

[52] Lee H, Han S, Kwon CS, Lee D. Biogenesis and regulation of the let-7 miRNAs and their functional implications. Protein & Cell. 2016;7:100–13. 10.1007/s13238-015-0212-y PMC4742387 26399619

[53] Seçkin E, Colinet D, Bailly-Bechet M, Seassau A, Bottini S, Sarti E, et al. Identification, evolutionary history and characteristics of orphan genes in root-knot nematodes. bioRxiv. 2025. doi: 10.64898/2025.12.19.695360

[54] Madeira F, Pearce M, Tivey ARN, Basutkar P, Lee J, Edbali O, et al. Search and sequence analysis tools services from EMBL-EBI in 2022. Nucleic Acids Res. 2022;50(W1):W276–9. doi: 10.1093/nar/gkac240 35412617 PMC9252731

[55] Trifinopoulos J, Nguyen L-T, von Haeseler A, Minh BQ. W-IQ-TREE: a fast online phylogenetic tool for maximum likelihood analysis. Nucleic Acids Res. 2016;44(W1):W232-5. doi: 10.1093/nar/gkw256 27084950 PMC4987875

[56] Blanc-Mathieu R, Perfus-Barbeoch L, Aury J-M, Da Rocha M, Gouzy J, Sallet E, et al. Hybridization and polyploidy enable genomic plasticity without sex in the most devastating plant-parasitic nematodes. PLoS Genet. 2017;13(6):e1006777. doi: 10.1371/journal.pgen.1006777 28594822 PMC5465968

[57] Taylor SC, Nadeau K, Abbasi M, Lachance C, Nguyen M, Fenrich J. The Ultimate qPCR Experiment: Producing Publication Quality, Reproducible Data the First Time. Trends Biotechnol. 2019;37(7):761–74. doi: 10.1016/j.tibtech.2018.12.002 30654913

[58] Hu W, DiGennaro PF. Identification of Suitable Meloidogyne spp. Housekeeping Genes. Journal of Nematology. 2019;51:e2019-55. doi: 10.21307/JOFNEM-2019-055 PMC6909023 34179799

[59] de Boer JM, Yan Y, Smant G, Davis EL, Baum TJ. In-situ Hybridization to Messenger RNA in Heterodera glycines. J Nematol. 1998;30(3):309–12. 19274224 PMC2620305

[60] Urwin PE, Lilley CJ, Atkinson HJ. Ingestion of double-stranded RNA by preparasitic juvenile cyst nematodes leads to RNA interference. Mol Plant Microbe Interact. 2002;15(8):747–52. doi: 10.1094/MPMI.2002.15.8.747 12182331

[61] Schindelin J, Arganda-Carreras I, Frise E, Kaynig V, Longair M, Pietzsch T, et al. Fiji: an open-source platform for biological-image analysis. Nat Methods. 2012;9(7):676–82. doi: 10.1038/nmeth.2019 22743772 PMC3855844

[62] Podolsky IA, Seppälä S, Xu H, Jin Y-S, O’Malley MA. A SWEET surprise: Anaerobic fungal sugar transporters and chimeras enhance sugar uptake in yeast. Metab Eng. 2021;66:137–47. doi: 10.1016/j.ymben.2021.04.009 33887459

