# Supplementary material for: The stage-specific regulation and role of root-knot nematode SWEET genes
Source: PLoS Pathog. 2026 May 6;22(5):e1014161. doi: 10.1371/journal.ppat.1014161 (PMC13148671; doi:10.1371/journal.ppat.1014161)
Supplement: S1 Table — Transcript per million of each discussed SWEET gene in M. incognita throughout development. (DOCX) [file ppat.1014161.s001.docx]

**S1 Table: *Meloidogyne incognita* SWEET TPM throughout development.** Transcript per million of each discussed SWEET gene in *M. incognita* throughout development.

| **Gene** | **Egg** | **J2** | **J3** | **Female** |
| --- | --- | --- | --- | --- |
| Minc_v4_contig_1g0004421 | 28.5 | 66.3 | 14.3 | 15.7 |
| Minc_v4_contig_5g0052531 | 9.7 | 20.2 | 5.1 | 5.8 |
| Minc_v4_contig_132g0513751 | 16.5 | 57.2 | 15.6 | 17.2 |
| Minc_v4_contig_133g0514781 | 5.9 | 36.4 | 9.5 | 7.4 |
| Minc_v4_contig_18g0172571 | 4.0 | 6.2 | 9.7 | 31.6 |
| Minc_v4_contig_94g0466131 | 6.2 | 10.3 | 19.8 | 90.5 |
| Minc_v4_contig_97g0471951 | 5.2 | 7.6 | 16.3 | 96.8 |
| Minc_v4_contig_49g0343631 | 0.1 | 0.1 | 0.0 | 21.0 |
| Minc_v4_contig_10g0094401 | 38.4 | 49.1 | 136.0 | 159.2 |
| Minc_v4_contig_48g0341891 | 17.8 | 15.5 | 47.2 | 60.8 |
