# Supplementary material for: The stage-specific regulation and role of root-knot nematode SWEET genes
Source: PLoS Pathog. 2026 May 6;22(5):e1014161. doi: 10.1371/journal.ppat.1014161 (PMC13148671; doi:10.1371/journal.ppat.1014161)
Supplement: S2 Table — Matrix generated by Clustal2.1 using amino acid sequences of the M. incognita orthologues of C. elegans SWEET genes. (DOCX) [file ppat.1014161.s002.docx]

**S2 Table: Percentage identity matrix of *M. incognita* SWEET genes.** Matrix generated by Clustal2.1 using amino acid sequences of the *M. incognita* orthologues of *C. elegans* SWEET genes.

| **SWEET** | ***M. incognita* orthologue** | **Sequence identity matrix** | | | | | | | | | |
| --- | --- | --- | --- | --- | --- | --- | --- | --- | --- | --- | --- |
| 7 | Minc_v4_contig_10g0094401 | **100** | 98.7 | 21.5 | 19.6 | 19.6 | 21.4 | 21.4 | 21.4 | 24.2 | 23.7 |
| 7 | Minc_v4_contig_48g0341891 | 98.7 | **100** | 21.9 | 19.6 | 19.6 | 21.0 | 21.0 | 21.0 | 24.6 | 24.2 |
| 5 | Minc_v4_contig_49g0343631 | 21.5 | 21.9 | **100** | 17.5 | 17.5 | 17.0 | 17.0 | 17.0 | 22.4 | 22.9 |
| 2 | Minc_v4_contig_1g0004421 | 19.6 | 19.6 | 17.5 | **100** | 97.4 | 31.5 | 31.7 | 31.7 | 33.5 | 33.5 |
| 2 | Minc_v4_contig_5g0052531 | 19.6 | 19.6 | 17.5 | 97.4 | **100** | 31.0 | 31.5 | 31.5 | 33.5 | 33.5 |
| 3 | Minc_v4_contig_18g0172571 | 21.4 | 21.0 | 17.0 | 31.5 | 31.0 | **100** | 99.5 | 99.3 | 33.2 | 33.2 |
| 3 | Minc_v4_contig_97g0471951 | 21.4 | 21.0 | 17.0 | 31.7 | 31.5 | 99.5 | **100** | 99.8 | 33.2 | 33.2 |
| 3 | Minc_v4_contig_94g0466131 | 21.4 | 21.0 | 17.0 | 31.7 | 31.5 | 99.3 | 99.8 | **100** | 33.2 | 33.2 |
| 4 | Minc_v4_contig_132g0513751 | 24.2 | 24.6 | 22.4 | 33.5 | 33.5 | 33.2 | 33.2 | 33.2 | **100** | 98.2 |
| 4 | Minc_v4_contig_133g0514781 | 23.7 | 24.2 | 22.9 | 33.5 | 33.5 | 33.2 | 33.2 | 33.2 | 98.2 | **100** |
