# Supplementary material for: The stage-specific regulation and role of root-knot nematode SWEET genes
Source: PLoS Pathog. 2026 May 6;22(5):e1014161. doi: 10.1371/journal.ppat.1014161 (PMC13148671; doi:10.1371/journal.ppat.1014161)
Supplement: S3 Table — Primers used to amplify DIG probes for in situ hybridisation and for qRT-PCR. (DOCX) [file ppat.1014161.s003.docx]

**S3 Table: *In situ* hybridisation probe and qRT-PCR primers.** Primers used to amplify DIG probes for *in situ* hybridisation and for qRT-PCR.

| **Gene** | **Forward** | **Reverse** | **Product size (bp)** | **Reference** |
| --- | --- | --- | --- | --- |
| Elongation factor 2 | CTCCTGGACACGTTGACTTC | GCACACAAACTCCTGAAACAC | 99 | Hu & DiGennaro, 2019 |
| *Mi-SWEET2* | AGTAGCAGGCAACGACGAAG | GGCCATAACCTCCACGGAAA | 163 | This study. |
| *Mi-SWEET3* | CTTTGCTGCACCGTTGAGTG | TGCCAAAAGACTGCCAATGC | 175 | This study. |
| *Mi-SWEET4* | TGCAACAATTGCTTCTCCACTT | GGGAAAGTTGCACTGAAGAAATGA | 197 | This study. |
| *Mi-SWEET5* | TCCTGGGAGGCTATATCCCTT | CCGAGAACGGCACACAGATA | 188 | This study. |
| *Mi-SWEET7* | AGCAGAAGGCTTTTCTTCCCT | ACGGCAGATAATTGGCCATACA | 198 | This study. |
