# Supplementary material for: The stage-specific regulation and role of root-knot nematode SWEET genes
Source: PLoS Pathog. 2026 May 6;22(5):e1014161. doi: 10.1371/journal.ppat.1014161 (PMC13148671; doi:10.1371/journal.ppat.1014161)
Supplement: S4 Table — Primers used in PCR to amplify Mi-HBL1 DIG probes for in situ hybridisation, as well as to amplify Mi-HBL1 and Mi-GAPDH for qRT-PCR and DIG probes. (DOCX) [file ppat.1014161.s004.docx]

**S4 Table: *Mi-HBL1* and *Mi-GAPDG* primers.** Primers used in PCR to amplify *Mi-HBL1* DIG probes for *in situ* hybridisation, as well as to amplify *Mi-HBL1* and *Mi-GAPDH* for qRT-PCR and DIG probes.

| **Gene** | **Forward** | **Reverse** | **Product size (bp)** | **Reference** |
| --- | --- | --- | --- | --- |
| *Mi-HBL1* | CATTGCGGGTTTAGCTGTGG | TCCAGCAGCCAATCTTTCCC | 162 | This study. |
| *Mi-GAPDH* | CGTGCAGCGGTTGAGAAGGA | GCGTCCGTGGGTGGAATCAT | 108 | This study. |
