# Supplementary material for: The stage-specific regulation and role of root-knot nematode SWEET genes
Source: PLoS Pathog. 2026 May 6;22(5):e1014161. doi: 10.1371/journal.ppat.1014161 (PMC13148671; doi:10.1371/journal.ppat.1014161)
Supplement: S1 Fig — The expression of the target genes were quantified by qRT-PCR using Elongation Factor 2 as a reference gene and displayed relative to nematode eggs for each gene. (DOCX) [file ppat.1014161.s005.docx]

**S1 Fig: qRT-PCR validation of RNA-seq expression profiles of ten *Meloidogyne incognita* SWEET genes.** The expression of the target genes were quantified by qRT-PCR using Elongation Factor 2 as a reference gene and displayed relative to nematode eggs for each gene.
